# Risk factors for bronchopulmonary dysplasia infants with respiratory score greater than four: a multi-center, prospective, longitudinal cohort study in China

**DOI:** 10.1038/s41598-023-45216-x

**Published:** 2023-10-19

**Authors:** Yan-ping Xu, Zheng Chen, Robert M. Dorazio, Guan-nan Bai, Li-zhong Du, Li-ping Shi

**Affiliations:** 1 NICU, Children’s Hospital, Zhejiang University School of Medicine and National Clinical Research Center for Child Health, 3333 Binsheng Road, Hangzhou, 310052 China; 2 Clinical Research Center, Children’s Hospital, Zhejiang University School of Medicine, Hangzhou, 310052 China; 3 Department of Child Health Care, Children’s Hospital, Zhejiang University School of Medicine and National Clinical Research Center for Child Health, Hangzhou, 310052 China

**Keywords:** Outcomes research, Paediatric research

## Abstract

Bronchopulmonary dysplasia (BPD) is the most common complication of prematurity involving both pre- and post-natal factors. A large, prospective, longitudinal cohort study was conducted to determine whether inflammation-related factors are associated with an increased risk of BPD in preterm infants who were born at a gestational age < 32 weeks, < 72 h after birth and respiratory score > 4. The study included infants from 25 participating hospitals in China between March 1, 2020 and March 31, 2022. The primary outcomes were BPD and severity of BPD at 36 weeks post-menstrual age. A total of 1362 preterm infants were enrolled in the study. After exclusion criteria, the remaining 1088 infants were included in this analysis, of whom, 588 (54.0%) infants were in the BPD group and 500 (46.0%) were in the non-BPD group. In the BPD III model, the following six factors were identified: birth weight (OR 0.175, 95% CI 0.060–0.512; *p* = 0.001), surfactant treatment (OR 8.052, 95% CI 2.658–24.399; *p* < 0.001), mean airway pressure (MAP) ≥ 12 cm H_2_O (OR 3.338, 95% CI 1.656–6.728; *p* = 0.001), late-onset sepsis (LOS) (OR 2.911, 95% CI 1.514–5.599; *p* = 0.001), ventilator-associated pneumonia (VAP) (OR 18.236, 95% CI 4.700–70.756; *p* < 0.001) and necrotizing enterocolitis (NEC) (OR 2.725, 95% CI 1.182–6.281; *p* = 0.019). Premature infants remained at high risk of BPD and with regional variation. We found that post-natal inflammation-related risk factors were associated with an increased risk of severe BPD, including LOS, VAP, NEC, MAP ≥ 12 cm H_2_O and use of surfactant.

## Introduction

Bronchopulmonary dysplasia (BPD) is the most common complication of prematurity. Data from major cohort studies reported the prevalence of BPD ranging from 11 to 50% owing to differences in gestational age (GA) or birth weight criteria for BPD diagnosis^1–5^. Despite advances in perinatal care that have improved survival rates, the incidence of BPD has remained constant or even increased, and a global burden of disease has reported a high mortality rate for infants with BPD^6^. Ventilator-associated lung injury has been reduced by the use of newer and gentler ventilation methods, but inflammation remains a key basis for pathogenesis.

Much remains to be learned about the role of inflammation in BPD. Studies have suggested that prenatal inflammation alter the development of fetal lungs, with consequences that can be harmful to premature newborns^5,7^. Infants exposed to inflammatory cascade may require respiratory support for a longer period of time and have a possible increased risk of BPD^8,9^. In addition, studies have shown that sepsis induces proinflammatory and profibrotic responses in the lungs of preterm infants, predisposing them to BPD^10^. In a hyperoxia-induced BPD rodent model, the extent of abnormal lung development is determined by the proportion and duration of oxygen supplementation^11^. When hyperoxia episodes are combined with hypoxic events that often occur in preterm infants, BPD pathology worsens and further promotes reactive oxygen species production and subsequent inflammation^12^. Together, these pathophysiological changes of systemic or lung inflammation promote fibrosis of the alveolar septum, damage pulmonary development, and eventually lead to the occurrence of BPD^13,14^.

Many cohort studies have comparatively evaluated the risk factors and outcomes associated with BPD infants^15–17^; however, the role of inflammation risk factors on the development of BPD remains unclear. Respiratory score for use in newborn infants with respiratory distress syndrome is proposed. In 1970, Downes et al. developed a scoring system for assessing respiratory distress in infants with respiratory distress syndrome and correlated it with blood gas parameters^18^. Downes’ respiratory scoring system is still in use; however, it has not been standardized for use in extremely preterm infants. Nevertheless, considering other clinical scoring systems, we chose the Downes scoring system because it is simple, noninvasive, inexpensive, and has prognostic value and good reliability and is used in clinical practice around the world^19–21^. The score is the sum of the 6 individual scores and is useful for tracking the severity of respiratory distress over time in a baby who is breathing spontaneously (Supplemental Table 1). Newborns scored on a scale of 0 to 2 for each component, and their total score ranged from 0 to 12, with 12 representing most severe distress. Higher scores were associated with an increased likelihood of needing advanced respiratory support^22,23^. We observed that other infants whose score remained less than 4 throughout the first 72 h of life were nearly always normal and rarely progresses to BPD from a clinical standpoint. Based on this, we hypothesize that inflammation plays a central role in the progression of BPD in the population with a score greater than 4 and that pre- and post-natal exposure to inflammation increases the risk of severe BPD. Given the need to better quantify the risks associated with exposure to inflammatory risk factors before and after birth, we examined this association in a population-based cohort study.

## Methods

### Ethical statement

The research protocol was approved by the Institutional Review Board (IRB) of Children’s Hospital, Zhejiang University School of Medicine (#2019-IRB-164). The trial was registered to Chinese Clinical Trial Registry under identifier ChiCTR2000030125 (23/02/2020). All methods were performed in accordance with the relevant guidelines and regulations. During hospitalization, written informed consents in view of prospective research of the clinical data were obtained for every included patient from their guardians, which took place from March 1, 2020, to March 31, 2022.

### Enrolled preterm infants

We conducted a multicenter, cohort study in level III and IV neonatal intensive care unit (NICU) in China. All enrolled preterm infants were born at a GA of < 32 weeks, < 72 h after birth with respiratory score > 4 at 25 participating hospitals (Supplemental methods). Approvals were obtained from the Ethics Committee at all participating centers. Exclusion criteria were congenital heart disease, congenital malformations of lung development, congenital digestive tract malformations, congenital renal malformations, congenital central nervous system malformations, congenital metabolic diseases, other congenital malformations, COVID-19 exposure and those whose BPD status were unavailable at 36-weeks postmenstrual age.

To investigate pre- and post-natal sepsis and other medical conditions with a similar systemic inflammatory response, we reviewed the following data for infants at risk of BPD: chorioamnionitis, antenatal antibiotics, antenatal corticosteroids, route of delivery, premature rupture of membranes (PROM), amniotic fluid contamination, GA, gender, birth weight, surfactant treatment, noninvasive respiratory support, invasive respiratory support, postnatal corticosteroids, intrauterine infection, EOS, LOS, central line associated bloodstream infections (CLABSIs), ventilator associated pneumonia (VAP), meningitis, necrotizing enterocolitis (NEC), extrauterine growth retardation (EUGR), patent ductus arteriosus (PDA), BPD and complications. A diagnosis of BPD was defined by a requirement of oxygen supplementation at 36 weeks postmenstrual age (PMA). The 2018 NICHD Workshop reclassified of severity based on modes of respiratory support grades (I, II, III and IIIA), a new category (IIIA) for early death (between 14 days of postnatal age and 36 weeks) owing to persistent parenchymal lung disease (Supplemental Table 2)^24^. Other diagnostic definitions on Supplemental methods.

### Prenatal exposures

Prenatal variables associated with a maternal or fetal pro-inflammatory state were chosen based on prior evidence^25^. These variables included PROM, chorioamnionitis and meconium-stained amniotic fluid (MSAF). Prolonged ROM was defined as rupture of the membranes ≥ 18 h prior to birth.

### Postnatal exposures

Postnatal variables associated with a neonatal pro-inflammatory state were limited to conditions that most often occur prior to development of BPD, which has a peak incidence of < 36 weeks’ postmenstrual gestational age in preterm infants^26^. Postnatal inflammatory-related variables included invasive respiratory support, mean airway pressure (MAP) ≥ 12cmH_2_O, non-invasive respiratory support, surfactant treatment, EOS, LOS, CLABSI, VAP, meningitis and NEC before 28 days after birth.

### Outcomes

To accomplish an analysis according to the principle of intention to identify risk factors, we defined our primary outcome as physiological BPD at 36 weeks’ postmenstrual age (PMA) and duration of respiratory support. Participants were assessed at 36 ± 1 weeks’ PMA and physiological BPD severity classified as the 2018 NICHD Workshop based on modes of respiratory support grades (Supplemental methods and Supplemental Table 2). Secondary outcomes were complications at the time of discharge and BPD occurrence in different regions.

### Statistical analysis

Data were collected and recorded using SPSS software (version 23.0) and the R programming language (https://www.R-project.org/). For all the statistical tests, a *p* < 0.05 was considered to be significant. Continuous parameters were expressed as the mean ± standard deviation or median (minimum–maximum) range. Categorical parameters were expressed as numbers (percentages), as appropriate. For comparisons of patients without and with BPD among different severity, one-way ANOVA testing or the Kruskal–Wallis test and chi square were adopted, as appropriate. Risk and protective factors for BPD were identified using a multilevel, multinomial logistic regression model that included random effects to account for regional differences in BPD. The performance of these factors in predicting BPD level was estimated using tenfold cross-validation to quantify the sensitivity and specificity of out-of-sample predictions of BPD level. A total of 100 random cross-validation samples were used to compute average values of sensitivity and specificity.

## Results

### Cohort selection and clinical characteristics of centers participating in the study

**Figure 1:**
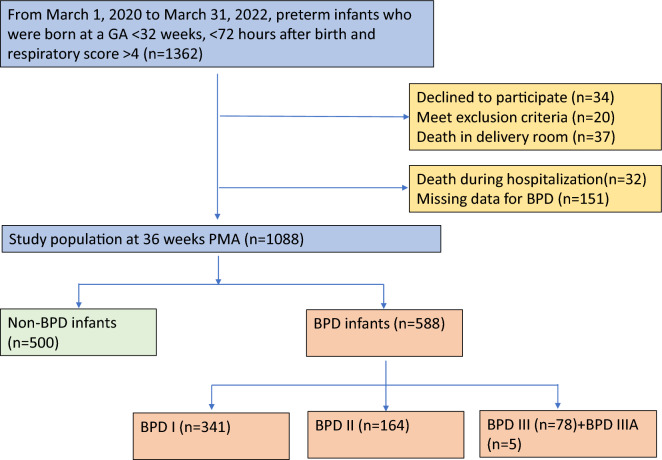
Study flow chart of 1362 preterm infants born at a gestational age < 32 weeks were enrolled in the study on their day < 72 h after birth. The remaining 1088 infants were included in this analysis, and of whom, 588 (54.0%) infants were in the BPD group, while 500 (46.0%) were in the non-BPD group. GA, gestational age; BPD, bronchopulmonary dysplasia.

**Figure 2:**
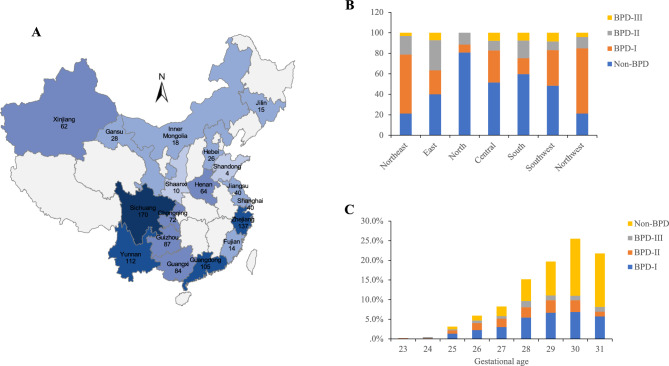
(**A**) Spatial distribution of 25 study sites in 18 provinces and municipalities directly under the central government across China. (**B**) Region distribution of BPD severity (% of Total). (**C**) Distribution of BPD severity at different gestational ages.

A total of 1362 inborn infants from 25 participating hospitals with an average of 30 beds in the neonatology departments and NICUs were included (Figs. 1, 2A). Twenty newborns were excluded because they met the exclusion criteria. Additionally, 34 infants whose parents declined to participate, 37 newborns who died in the delivery room, 32 newborns who died during hospitalization, and 151 newborns who had missing initial respiratory management data were excluded. The remaining 1088 newborns were included in this analysis, and of whom, 588 (54.0%) were in the BPD group, and 500 (46.0%) were in the non-BPD group (Fig. 1). When the population was stratified by BPD severity, then I, II, III and IIIA BPD accounted for 31.3% (341/1088), 15.1% (164/1088), 7.2% (78/1088) and 0.46% (5/1088) of the sample. The incidence of BPD was highest in Northwest and Northeast region of China (Fig. 2B). The final cohort had a median (range) birthweight of 1.27 (0.55–2.44) kg and a GA of 29 (23–31) weeks, respectively. There were 617 males (56.7%) and 471 females (43.3%). The newborn characteristics are shown in Table 1.

**Table 1:** Characteristics of the study population (n = 1088).

Characteristics	Value
Gestational age(weeks), median (range)	29 (23–31)
Birth weight, median (range), kg	1.27 (0.55–2.44)
Male, n (%)	617 (56.7)
Region	
Northeast	33 (3.0)
East	235 (21.6)
North	26 (2.4)
Central	64 (5.9)
South	189 (17.4)
Southwest	441 (40.5)
Northwest	100 (9.2)
Chorioamnionitis	89 (8.2)
Antenatal antibiotics, n (%)	336 (30.9)
Antenatal steroids, n (%)	790 (72.6)
Route of delivery, CS, n (%)	618 (56.8)
PPROM, n (%)	407 (37.4)
Duration rupture of membranes, n (%)	
< 18 h	127 (11.7)
≥ 18 h	274 (25.2)
MSAF	77 (7.1)
Surfactant	775 (71.2)
SGA	85 (7.8)
Postnatal steroids, n (%)	348 (32.0)
Invasive respiratory support, n (%)	593 (54.5)
Duration invasive, median (range), days	1 (0–88)
MAP ≥ 12cmH_2_O	228 (21.0)
Duration MAP ≥ 12cmH_2_O, median (range), days	0 (0–68)
Non-invasive respiratory support, n (%)	1021 (93.8)
Duration non-invasive, median (range), days	14 (0–118)
EOS	224 (20.6)
LOS	267 (24.5)
CLABSI	37 (3.4)
VAP	78 (7.2)
Meningitis	55 (5.1)
NEC	115 (10.6)
BPD	588 (54.0)
I	341 (31.3)
II	164 (15.1)
III	78 (7.2)
IIIA	5 (0.5)
PDA	302 (27.8)
PDA surgery	16 (1.5)
EUGR	179 (16.5)
PH	54 (5.0)
IVH(III/IV)	98 (9.0)
PVL	38 (3.5)
ROP	244 (22.4)
Total days in hospital	50 (12–187)

CS, cesarean section; PPROM, preterm premature rupture of membranes; MSAF, meconium-stained amniotic fluid; SGA, small for gestational age; MAP, mean airway pressure; EOS, early-onset sepsis; LOS, late-onset sepsis; CLABSI, central line associated bloodstream infections; VAP, ventilator-associated pneumonia; NEC, necrotizing enterocolitis; BPD, bronchopulmonary dysplasia; PDA, patent ductus arteriosus; EUGR, extrauterine growth retardation; PH, pulmonary hypertension; IVH, intraventricular hemorrhage; PVL, periventricular leukomalacia; ROP, retinopathy of prematurity.

The GA distribution of preterm infants was as follows: 30 and 31 weeks accounted for 504/1072 (47.0%), 28 and 29 weeks accounted for 374/1072 (34.9%), and < 28 weeks accounted for 18.1% (194/1072), indicating a skewed distribution. The incidence of BPD in preterm infants was significantly higher at lower GAs. For example, at GAs of 31 weeks and 30 weeks, the incidence of BPD was 40.5% (204/504). Whereas a significantly higher incidence of 59.6% (223/374) was observed at 29- and 28-weeks’ GAs. The highest incidence was 76.3% (148/194) at < 28 weeks GAs (Fig. 2C). The birth weights of 1079 preterm infants (9/1088 missing birthweight data) were mainly distributed between 1000 and 1499 g. More specifically, 24.9% (269/1079) were ≥ 1500 g, 54.3% (586/1079) were 1000-1499 g, and 20.8% (224/1079) were < 1000 g. The incidence of BPD in preterm infants was significantly higher among infants with lower birth weight, especially for extremely low birth weight (ELBW) infants. The incidence of BPD in ELBW infants was as high as 75.0% (168/224), and in very low birth weight (VLBW) infants (birth weight 1499–1000 g) the incidence was 54.6% (320/586). The incidence of BPD in infants with a birth weight ≥ 1500 g was only 36.1% (97/269). The patients with BPD were mainly ELBW and VLBW.

Maternal PROM, MSAF, use of surfactant, invasive respiratory support, mean airway pressure (MAP) ≥ 12 cm H_2_O, non-invasive respiratory support, EOS, LOS, VAP, meningitis, NEC, and PDA were each higher in the BPD group than in the non-BPD group (Table 2). There were no significant differences in gender, chorioamnionitis, antenatal steroids, SGA, and CLABSIs among the groups. Preterm complications, such as EUGR, PH, IVH, PVL and ROP, were significantly higher in the BPD groups than in the non-BPD group (Table 2).

**Table 2:** Comparisons between patients with and without BPD.

	Non-BPD (n = 500)	BPD-I (n = 341)	BPD-II (n = 164)	BPD-III (n = 78)	BPD (n = 583) + BPD IIIA (n = 5)	*P* value*	*P* value^#^
	Value	Value	Value	Value	Value		
Gestational age(weeks), median (range)	30 (25–31)	29 (23–31)	28 (23–31)	29 (24–31)	29 (23–31)	< 0.001	< 0.001
Birth weight, median (range), kg	1.39 (0.70–2.44)	1.20 (0.64–1.97)	1.10 (0.56–1.81)	1.08 (0.55–2.05)	1.18 (0.55–2.05)	< 0.001	< 0.001
Region							
Northeast	7 (1.4)	19 (5.6)	6 (3.7)	1 (1.3)	26 (4.4)^a^	< 0.001	< 0.001
East	94 (18.8)	55 (16.1)	69 (42.1)	17 (21.8)	141 (24.0)^a^		
North	21 (4.2)	2 (0.6)	3 (1.8)	0 (0.0)	5 (0.9)^a^		
Central	33 (6.6)	20 (5.9)	6 (3.7)	5 (6.4)	31 (5.3)		
South	111 (22.2)	29 (8.5)	32 (19.5)	14 (17.9)	78 (13.3)^a^		
Southwest	213 (42.6)	153 (44.9)	37 (22.6)	37 (47.4)	228 (38.8)		
Northwest	21 (4.2)	63 (18.5)	11 (6.7)	4 (5.1)	79 (13.4)^a^		
Male, n (%)	280 (56.5)	196 (57.8)	90 (54.9)	49 (63.6)	337 (57.3)	0.609	0.374
Chorioamnionitis	35 (7.0)	35 (10.3)	13 (7.9)	5 (6.4)	54 (9.2)	0.354	0.190
Antenatal steroids, n (%)	371 (74.2)	238 (69.8)	122 (74.4)	54 (69.2)	419 (71.3)	0.442	0.278
Route of delivery, CS, n (%)	298 (60.1)	187 (55.0)	86 (52.8)	44 (56.4)	320 (54.4)	0.298	0.070
PPROM, n (%)	211 (42.6)	119 (35.3)	51 (31.3)	22 (28.2)	196 (33.3)	0.008	0.002
MSAF	23 (4.7)	28 (8.3)	21 (12.9)	5 (6.5)	54 (9.2)	0.005	0.004
Surfactant	273 (54.6)	271 (79.9)	153 (93.3)	73 (93.6)	502 (85.4)	< 0.001	< 0.001
SGA	31 (6.3)	29 (8.6)	14 (8.8)	11 (14.7)	54 (9.2)	0.084	0.069
Postnatal steroids, n (%)	82 (16.4)	115 (34.0)	101 (62.0)	47 (61.8)	266 (45.2)	< 0.001	< 0.001
Invasive respiratory support, n (%)	198 (40.5)	196 (57.8)	132 (81.5)	62 (82.7)	395 (67.2)	< 0.001	< 0.001
Duration invasive, median (range), days	0 (0–88)	2 (0–55)	6 (0–69)	19 (0–73)	4 (0–88)	< 0.001	< 0.001
MAP ≥ 12cmH_2_O	43 (9.2)	82 (24.8)	62 (38.5)	38 (50.0)	185 (31.5)	< 0.001	< 0.001
Non-invasive respiratory support, n (%)	461 (94.1)	331 (97.9)	151 (93.2)	73 (96.1)	560 (95.2)	0.036	0.075
Duration non-invasive, median (range), days	9 (0–74)	18 (0–63)	27 (0–83)	25 (0–118)	20 (0–118)	< 0.001	< 0.001
EOS	63 (12.7)	84 (24.8)	50 (30.9)	26 (33.3)	161 (27.4)	< 0.001	< 0.001
LOS	67 (13.6)	114 (34.0)	50 (31.1)	34 (43.6)	198 (33.7)	< 0.001	< 0.001
CLABSI	11 (2.2)	15 (4.4)	5 (3.1)	6 (7.7)	26 (4.4)	0.057	< 0.001
VAP	3 (0.6)	24 (7.1)	24 (15.0)	25 (32.5)	75 (12.8)	< 0.001	< 0.001
Meningitis	15 (3.0)	23 (6.8)	10 (6.2)	7 (9.1)	40 (6.8)	0.024	0.004
NEC	29 (5.8)	35 (10.3)	33 (20.1)	17 (21.8)	86 (14.6)	< 0.001	< 0.001
PDA	94 (18.9)	118 (34.8)	58 (35.8)	31 (40.8)	208 (35.4)	< 0.001	< 0.001
EUGR	49 (9.8)	61 (17.9)	36 (22.0)	32 (41.0)	130 (22.1)	< 0.001	< 0.001
PH	8 (1.6)	21 (6.2)	12 (7.3)	12 (15.4)	46 (7.7)	< 0.001	< 0.001
IVH(III/IV)	5 (1.0)	61 (17.9)	22 (13.4)	9 (11.5)	93 (15.8)	< 0.001	< 0.001
PVL	6 (1.2)	16 (4.7)	11 (6.7)	5 (6.4)	32 (5.4)	0.001	< 0.001
ROP	79 (15.8)	76 (22.3)	56 (34.1)	31 (39.7)	165 (28.1)	< 0.001	< 0.001
Total days in hospital, median (range), days	43 (12–102)	55 (25–115)	66 (31–167)	75 (31–187)	60 (20–187)	< 0.001	< 0.001

*For comparisons of infants without and with BPD among different severity, Kruskal–Wallis test and chi square were adopted, as appropriate.

^#^For comparisons of infants non-BPD and with BPD, Mann–Whitney U test and chi square were adopted, as appropriate. ^a^*p* < 0.05.

CS, cesarean section; PPROM, preterm premature rupture of membranes; MSAF, meconium-stained amniotic fluid; SGA, small for gestational age; MAP, mean airway pressure; EOS, early-onset sepsis; LOS, late-onset sepsis; CLABSI, central line associated bloodstream infections; VAP, ventilator-associated pneumonia; NEC, necrotizing enterocolitis; BPD, bronchopulmonary dysplasia; PDA, patent ductus arteriosus; EUGR, extrauterine growth retardation; PH, pulmonary hypertension; IVH, intraventricular hemorrhage; PVL, periventricular leukomalacia; ROP, retinopathy of prematurity.

### Multinomial logistic regression analysis of risk factors for BPD in preterm infants

**Figure 3:**
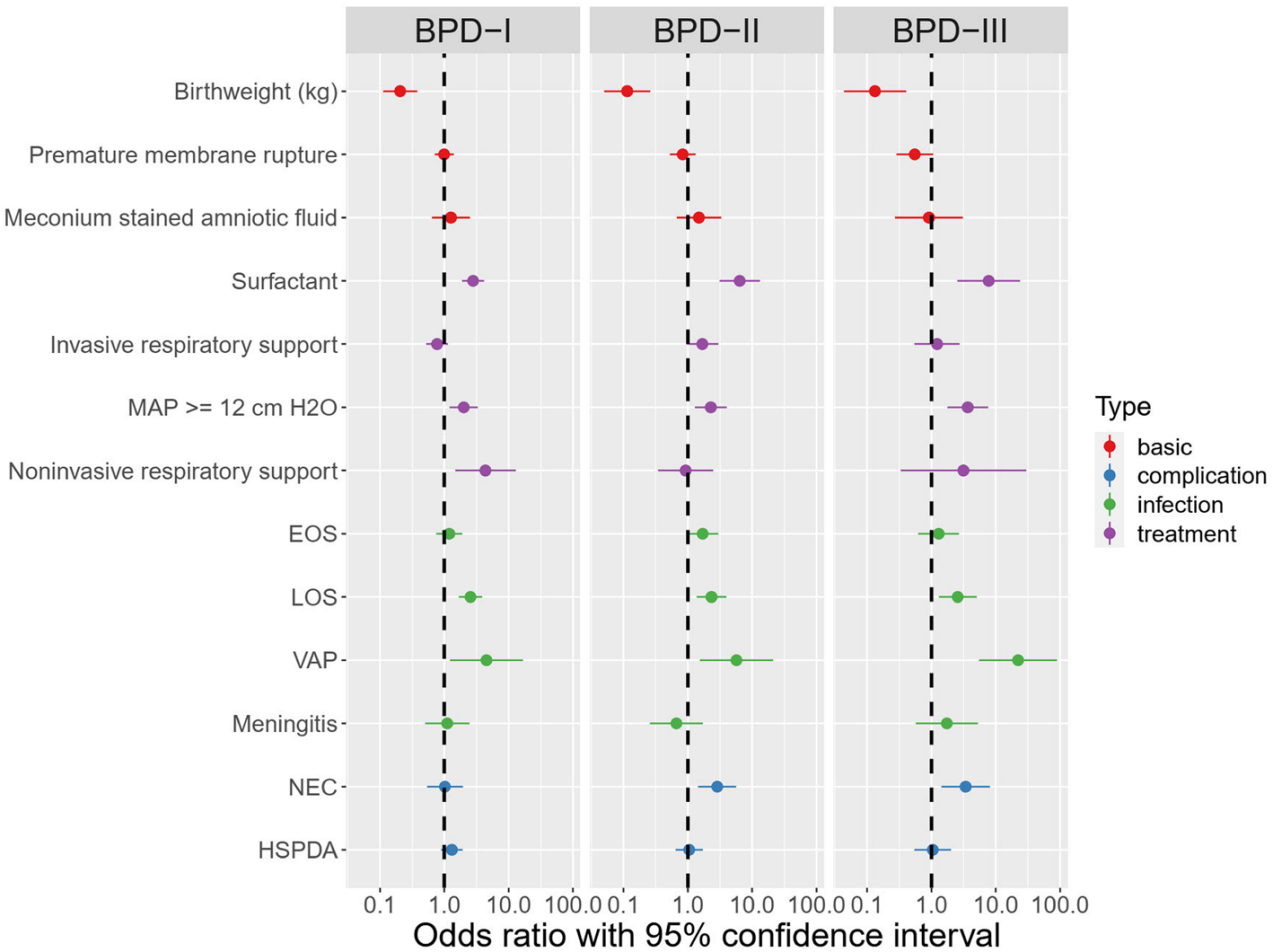
The forest plot of OR for the different severity of BPD infants.

Variables that were significantly different (at a level of *p* < 0.05) were used as predictors of BPD level in a multilevel, multinomial logistic regression model. These variables included birthweight, PROM, MSAF, surfactant treatment, invasive respiratory support, MAP ≥ 12 cm H_2_O, non-invasive respiratory support, EOS, LOS, VAP, meningitis, NEC, and PDA. Six factors were informative of BPD I as indicated by their odds ratios (ORs): birth weight (OR 0.239, 95% CI 0.133–0.430; *p* < 0.001), surfactant treatment (OR 3.163, 95% CI 2.154–4.646; *p* < 0.001), MAP ≥ 12cmH_2_O (OR 2.077, 95% CI 1.272–3.392; *p* = 0.003), non-invasive respiratory support (OR 3.743, 95% CI 1.337–10.480; *p* = 0.012), LOS (OR 2.857, 95% CI 1.895–4.308; *p* < 0.001), and VAP (OR 4.133, 95% CI 1.139–14.992; *p* < 0.001). Seven factors were identified for BPD II: birth weight (OR 0.101, 95% CI 0.045–0.226; *p* < 0.001), use surfactant (OR 6.188, 95% CI 3.052–12.546; *p* < 0.001), invasive respiratory support (OR 1.843, 95% CI 1.057–3.215; *p* = 0.031), MAP ≥ 12 cm H_2_O (OR 2.339, 95% CI 1.344–4.069; *p* = 0.003), LOS (OR 2.077, 95% CI 1.230–3.508; *p* = 0.006), VAP (OR 5.816, 95% CI 1.585–21.343; *p* = 0.008), and NEC (OR 2.772, 95% CI 1.423–5.397; *p* = 0.003). Six factors were identified for BPD III: birth weight (OR 0.175, 95% CI 0.060–0.512; *p* = 0.001), surfactant treatment (OR 8.052, 95% CI 2.658–24.399; *p* < 0.001), MAP ≥ 12 cm H_2_O (OR 3.338, 95% CI 1.656–6.728; *p* = 0.001), LOS (OR 2.911, 95% CI 1.514–5.599; *p* = 0.001), and VAP (OR 18.236, 95% CI 4.700–70.756; *p* < 0.001), and NEC (OR 2.725, 95% CI 1.182–6.281; *p* = 0.019). PROM, MSAF, EOS, meningitis, and PDA were not useful in distinguishing between the BPD groups and the non-BPD group. A complete overview of the fitted model is shown in Fig. 3 and Table 3. Our analysis based on cross-validation sampling showed that the regression model’s predictions of BPD I had a sensitivity of 69% and a specificity of 71%. The regression model’s predictions of BPD II had a sensitivity of 81% and a specificity of 76%, and the predictions of BPD III were similar (sensitivity of 80% and specificity of 81%) (Table 4).

**Table 3:** Multivariate logistic regression analysis of the determinants of BPD (n = 1083).

	BPD-I (n = 341)	BPD-II (n = 164)	BPD-III (n = 78)
Birth weight (kg)	0.239 (0.133–0.430)**	0.101 (0.045–0.226)**	0.175 (0.060–0.512)**
PPROM	0.941 (0.675–1.311)	0.861 (0.547–1.356)	0.652 (0.345–1.234)
MSAF	1.247 (0.639–2.435)	1.639 (0.749–3.591)	0.926 (0.279–3.071)
Surfactant	3.163 (2.154–4.646)**	6.188 (3.052–12.546)**	8.052 (2.658–24.399)**
Invasive respiratory support	0.739 (0.502–1.088)	1.843 (1.057–3.215)*	1.167 (0.522–2.605)
MAP ≥ 12cmH_2_O	2.077 (1.272–3.392)**	2.339 (1.344–4.069)**	3.338 (1.656–6.728)**
Non-invasive respiratory support	3.743 (1.337–10.480)*	1.086 (0.426–2.771)	5.233 (0.612–44.742)
EOS	1.378 (0.880–2.159)	1.369 (0.802–2.337)	1.350 (0.674–2.707)
LOS	2.857 (1.895–4.308)**	2.077 (1.230–3.508)**	2.911 (1.514–5.599)**
VAP	4.133 (1.139–14.992)*	5.816 (1.585–21.343)**	18.236 (4.700–70.756)**
Meningitis	0.951 (0.434–2.081)	0.829 (0.325–2.114)	1.409 (0.479–4.142)
NEC	1.142 (0.616–2.119)	2.772 (1.423–5.397)**	2.725 (1.182–6.281)*
PDA	1.317 (0.903–1.922)	1.062 (0.656–1.717)	1.008 (0.529–1.922)

*p < 0.05; **p < 0.01; PPROM, preterm premature rupture of membranes; MSAF, meconium-stained amniotic fluid; MAP, mean airway pressure; EOS, early-onset sepsis; LOS, late-onset sepsis; VAP, ventilator-associated pneumonia; NEC, necrotizing enterocolitis; BPD, bronchopulmonary dysplasia; PDA, patent ductus arteriosus.

**Table 4:** Estimated performance of predictors of BPD.

	AUC	Sensitivity	Specificity
BPD-I	0.75	0.69	0.71
BPD-II	0.84	0.81	0.76
BPD-III	0.87	0.80	0.81

## Discussion

Our study found that the total incidence of BPD in preterm infants was 54.0%. The incidence of BPD is higher than that reported in the literature. However, our study included whose respiratory score was greater than 4 points, which definitely had a higher prevalence of BPD than those whose respiratory score was not included as a condition due to the increased likelihood of needing advanced respiratory support. Stratified analysis by geographic region showed that the incidence of BPD in preterm infants varied greatly among different geographic regions, with the highest incidence in the Northwest and Northeast in China, which may be related to the economic inequality and cultural level. Natural geographical environment factors in China's are very different, the distribution of resources is extremely unbalanced. In addition, the eastern region, especially the coastal areas, has extensive connections with countries around the world, a high level of urbanization, and relatively perfect infrastructure such as transportation and communication. Due to these differences, the spatial distribution of medical resources in different regions of China is also different. Despite these factors, we found few published studies investigating socioeconomic differences in preterm care and predictors of complications associated with preterm birth. The lack of standardization of care for preterm infants in different hospitals may have implications for the management of BPD and preterm-related complications. The homogenized management of preterm infants is needed and it can guide care optimization and improve long-term outcomes for high-risk, understudied infants. Genetic variants may contribute to BPD risk or the susceptibility to some perinatal complications possibly associated with increased need for ventilatory support and/or lung damage^27^, but genetic background of BPD has not been fully verified. Despite the known role of coding proteins in the pathogenesis of the moderate to severe BPD, some results regarding the association between genetic polymorphisms and risk factors for BPD are negative^28^. Moreover, most of data were collected in small sample studies, their statistical power is low. An increase in the number of patients and meta-analysis of published data may overcome this problem, but it is difficult to carry out existing data pools due to the heterogeneity of infants. A large population-based, observational study reported in developed countries, infants who survived to 36 weeks’ PMA had a BPD incidence of 49.8% (4307/8641), but the incidence of BPD exceeded 75% in infants who were 22–24 weeks’ GA^29^. The results of the present study showed that the lower the GA and birth weight, the greater the risk of BPD, which is consistent with the results of many studies at home and abroad^30–32^.

Our study suggests that several postnatal inflammation-related risk factors, including LOS, VAP, NEC, MAP ≥ 12cmH_2_O and surfactant treatment were also associated with an increased risk of BPD. A recent retrospective study involving VLBW infants at 24–28 weeks of gestation showed that LOS was risk factors for BPD at 25–28 weeks of gestation, and effective postpartum resuscitation was the most important factor determining the severity of BPD at 24 weeks gestation^16^. The incidence of BPD in preterm infants with intrauterine infection is low, while neonatal sepsis after birth is significantly increased, and the pathogenic bacteria have a regulating effect on the occurrence of BPD^33^. Cokyaman et al. conducted a retrospective study involving 872 VLBW infants to determine the relationship between the frequency of BPD, perinatal risk factors and other prematurity comorbidities. LOS was shown to affect the development of BPD (OR 2.7; 95% CI 1.6–4.5; *p* < 0.01); however, there was a significant, but non-linear risk relationship between LOS and BPD^34^. Based on the multivariate analysis, LOS was a risk factors for BPD, two or more episodes of LOS were significantly associated with BPD and severe BPD. Our study also showed that NEC is associated with an increased risk of BPD. Inflammatory processes, such as NEC and LOS, may increase the risk of BPD development through direct and indirect mechanisms. Proinflammatory cytokines may have direct effects on lung development^35,36^. On the other hand, children with NEC and LOS tend to require more aggressive and prolonged mechanical ventilation, which may lead to increased lung injury^35,36^.

VAP is associated with significant morbidity owing to prolonged requirement of ventilation and an increased risk of a subsequent episode of BPD. Wang et al. reported that 25.4% of neonatal VAP episodes were polymicrobial VAP episodes, which were more likely to occur in neonates with long-term intubation and underlying chronic comorbidities, especially BPD^37^. In our study infants with VAP was also confirmed to increase the risk of BPD. In our multi-center study, the utilization rate of invasive ventilator in Northeast was the highest, and the duration of ventilator in Northeast and Northwest were the longest. Meanwhile, requiring surfactant therapy was most in Northeast and Northwest, which may be related to the severity of early RDS and may also be related to the limitations of early respiratory management^38^. The regression model with postnatal surfactant as a risk factor showed that it had a significant effect on BPD. Owing to the strong correlation between postnatal surfactants and RDS severity, RDS was not included in the model. During BPD development, most infants initially experience RDS, and many are given surfactants after birth. Therefore, a secondary trigger of BPD in preterm infants can be said to be severe RDS^34^. Higher airway pressure can reduce oxygen requirement in premature infants who need respiratory support. Among premature infants who are given respiratory support at birth, the peak inspiratory ventilator pressure is a predictor of BPD^39^. Under higher airway pressure, it is easier to cause pulmonary barotrauma, to trigger an inflammatory reaction, and develop BPD.

The risk factors in our analyses were proxies for inflammation, and not specific inflammatory mediators themselves. There are likely numerous inflammatory factors and mechanisms by which inflammation influence BPD development. Our study was limited by examining a relatively narrow and non-specific set of factors. In summary, premature infants with a GA < 32 weeks remain at high risk of BPD with geographic regional variation. We identified postnatal inflammation-related risk factors associated with an increased risk of BPD, including evolving LOS, VAP, NEC, MAP ≥ 12cmH_2_O, and surfactant treatment. It has been suggested that avoidance of postnatal infections reduces inflammation in the developing lungs more than avoidance of invasive mechanical ventilation.

## Ethical approval

The research protocol was approved by the Institutional Review Board (IRB) of Children’s Hospital, Zhejiang University School of Medicine (approval number, 2019-IRB-164). The trial was registered to Chinese Clinical Trial Registry under identifier ChiCTR2000030125. During hospitalization, written informed consents in view of prospective research of the clinical data were obtained for every included patient from their guardians, which took place from March 1, 2020, to March 31, 2022.

### Supplementary Information

Supplementary Information 1.

Supplementary Information 2.

Supplementary Information 3.

## Data Availability

The data that support the findings of this study are available from the corresponding author, upon reasonable request.
