# Supplementary material for: Risk factors for bronchopulmonary dysplasia infants with respiratory score greater than four: a multi-center, prospective, longitudinal cohort study in China
Source: Sci Rep. 2023 Oct 19;13:17868. doi: 10.1038/s41598-023-45216-x (PMC10587148; doi:10.1038/s41598-023-45216-x)
Supplement: Supplementary file 1 — Supplementary Information 1. [file 41598_2023_45216_MOESM1_ESM.docx]

**Supplemental Methods**

**Participating hospitals**

Chengdu Women's & Children's Central Hospital, Qujing Maternity & Child Healthcare Hospital, Shenzhen Maternity & Child Healthcare Hospital, Guiyang Maternity & Child Healthcare Hospital, Ningbo Women & Children's hospital, The First Affiliated Hospital of Xinjiang Medical University, Children's Hospital Zhejiang University School of Medicine, Guangxi Yulin Maternity & Child Healthcare Hospital, Children’s Hospital of Chongqing Medical University, Women’s Hospital of Nanjing Medical University, The First Affiliated Hospital of Guangxi Medical University, Henan Provincial People's Hospital, Gansu Provincial Maternity and Child-care Hospital, Bethune International Peace Hospital of People's Liberation Army of China, Henan Children’s Hospital, Chongqing Health Center for Women and Children, Shanghai Children's Medical Center, Children’s Hospital Affiliated to Shanghai Jiao Tong University School of Medicine, Inner Mongolia People's Hospital, The First Hospital of Jilin University, Quanzhou Women's and Children's Hospital, Shaanxi Provincial People's Hospital, The Second Affiliated Hospital of Guangxi Medical University, Kunming Children's Hospital, Taian Maternity and Child Health Hospital.

**Definitions**

China is generally divided into seven regions: Northeast, East, North, Central, South, Southwest and Northwest. Northeast includes Heilongjiang, Jilin, Liaoning, eastern Inner Mongolia. In East China, they are Shanghai, Jiangsu, Zhejiang, Anhui, Fujian, Jiangxi, Shandong and Taiwan. North China refers to Beijing, Tianjin, Hebei and Shanxi provinces, and the central part of Inner Mongolia. Central China, including Henan, Hubei and Hunan provinces. South China includes Guangdong, Guangxi, Hainan, Hong Kong, Macao. In southwest China, Sichuan, Guizhou, Yunnan, Tibet and Chongqing are five provinces and municipalities. Northwest region includes Shaanxi, Gansu, Qinghai, Ningxia, Xinjiang five provinces and autonomous regions. PROM is defined as rupture of membranes before the onset of labor^1^. Definitions of chorioamnionitis variability included some degree of maternal fever plus at least 2 clinical risk factors, such as maternal or fetal tachycardia, maternal leukocytosis, uterine tenderness, or purulent cervical discharge^2^. Small for gestational age (SGA) refers to birthweight below the 10^th^ percentile for GA^3^. EUGR was defined as the tenth percentile of weight below the growth curve at the corrected GA of 36 weeks. EOS is defined as the onset of systemic signs of infection before 7 days of age and LOS is defined as the onset of systemic signs of infection at ≥7 days of age^4^. The diagnosis of VAP in intubated preterm infants is highly challenging. The Centers for Disease Control and Prevention (CDC) guidelines employ the same VAP definition for all infants <1 year of age and include clinical criteria (available on line at <https://www.cdc.gov/nhsn/pdfs/pscmanual/6pscvapcurrent.pdf>). Instead, in the NICU setting, we rely on semi-quantitative neutrophil counts and quantitative bacterial culture results from tracheal aspirate fluids in conjunction with clinical and radiographic signs to diagnose VAP^5^. IVH grade III-IV was IVH with ventricular enlargement or parenchymal hemorrhage, as classified according to the Papile system^6^; The following diagnoses referenced Practice of neonatology (Fifth Edition)^7^: NEC, periventricular leukomalacia (PVL), PDA, pulmonary hypertension (PH), and retinopathy of prematurity (ROP).

**Non-Authors’ information**

Supplemental Table 3. Participating centers and investigators of the BPD cohort study group.

**Reference**

1 Dayal, S. & Hong, P. L. in *StatPearls* (2022).

2 Tita, A. T. & Andrews, W. W. Diagnosis and management of clinical chorioamnionitis. *Clin Perinatol* **37**, 339-354, doi:10.1016/j.clp.2010.02.003 (2010).

3 Battaglia, F. C. & Lubchenco, L. O. A practical classification of newborn infants by weight and gestational age. *J Pediatr* **71**, 159-163, doi:10.1016/s0022-3476(67)80066-0 (1967).

4 American Academy of Pediatrics. Group B streptococcal infections. In: Red Book: 2018 Report of the Committee on Infectious Diseases, 31st ed, Kimberlin DW, Brady MT, Jackson MA, Long SS (Eds), American Academy of Pediatrics, Itasca, IL 2018. p.762.

5 Ergenekon, E. & Cataltepe, S. Ventilator-associated pneumonia in the NICU: time to boost diagnostics? *Pediatr Res* **87**, 1143-1144, doi:10.1038/s41390-019-0672-5 (2020).

6 Papile, L. A., Burstein, J., Burstein, R. & Koffler, H. Incidence and evolution of subependymal and intraventricular hemorrhage: a study of infants with birth weights less than 1,500 gm. *J Pediatr* **92**, 529-534, doi:10.1016/s0022-3476(78)80282-0 (1978).

7 Shao XM, Ye HM, Qiu XS. Practice of neonatology Edition 5[M]. People’s Medical Publishing House; 2019
