# Supplementary material for: Risk factors for bronchopulmonary dysplasia infants with respiratory score greater than four: a multi-center, prospective, longitudinal cohort study in China
Source: Sci Rep. 2023 Oct 19;13:17868. doi: 10.1038/s41598-023-45216-x (PMC10587148; doi:10.1038/s41598-023-45216-x)
Supplement: Supplementary file 2 — Supplementary Information 2. [file 41598_2023_45216_MOESM2_ESM.docx]

| **Supplemental Table 1. The Respiratory Score** |  |  |  |
| --- | --- | --- | --- |
| **Score** | **0** | **1** | **2** |
| **Respiratory rate** | 40 to 60/minute | 60 to 80/minute | >80/minute |
| **Oxygen requirement** | None | ≤50% | > 50% |
| **Retractions** | None | Mild to moderate | Severe |
| **Grunting** | None | With stimulation | Continuous at rest |
| **Breath sounds on auscultation** | Easily heard throughout | Decreased | Barely heard |
| **Prematurity** | >34 weeks | 30 to 34 weeks | <30 weeks |
| A baby receiving oxygen prior to the setup of an oxygen of an oxygen analyzer should be assigned a score of 1 | | | |
| Adapted from Downes JJ, Vidyasagar D, Boggs TR, Jr., Morrow GM, 3rd. Respiratory distress syndrome of newborn infants. I. New clinical scoring system (RDS score) with acid--base and blood-gas correlations. Clin Pediatr (Phila). 1970;9(6):325-31. | | | |

| **Supplemental Table 2. 2018 NICHD Workshop criteria based on oxygen concentration** | | | | | |
| --- | --- | --- | --- | --- | --- |
| **Grades** | **Invasive IPPV** | **nCPAP, NIPPV, or nasal cannula >=3L/min** | **Nasal cannula flow of 1 to <3L/min** | **Nasal cannula flow of <1L/min** | **Hood O2** |
| I (mild) | - | 21 | 22 to 29 | 22 to 70 | 22 to 29 |
| II (moderate) | 21 | 22 to 29 | ≥30 | ≥70 | ≥30 |
| III (severe) | >21 | ≥30 | - | - | - |
| III (A) | - | - | - | - | - |

Higgins RD, Jobe AH, Koso-Thomas M, Bancalari E, Viscardi RM, Hartert TV, et al. Bronchopulmonary Dysplasia: Executive Summary of a Workshop. J Pediatr. 2018 Jun;197:300-08
